# Disturbed Sleep Among Adolescents Living in 2 Communities on the Texas-Mexico Border, 2000-2003

**Published:** 2010-02-15

**Authors:** Adriana Pérez, Robert E. Roberts, Maureen Sanderson, Belinda Reininger, Maria Isabel Aguirre-Flores

**Affiliations:** University of Texas Health Science Center at Houston. When this article was written, Dr Pérez was affiliated with the University of Louisville, Louisville, Kentucky. All work was performed at the University of Texas Health Science Center at Houston, Brownsville, Texas.; University of Texas Health Science Center at Houston, Houston, Texas; Meharry Medical College, Nashville, Tennessee; University of Texas Health Science Center at Houston and Hispanic Health Research Center at the Lower Rio Grande Valley, Brownsville, Texas; Secretaría de Educación de Tamaulipas, Ciudad Victoria

## Abstract

**Introduction:**

Disturbed sleep is a public health problem, but few studies describe the prevalence of sleep problems among Hispanic adolescents. We estimated the prevalence of disturbed sleep and associated factors among ninth graders living on the Texas-Mexico border.

**Methods:**

We used probabilistic sampling to conduct 2 cross-sectional, school-based surveys: 1 during the 2000-2001 school year in the Lower Rio Grande Valley, Texas (n = 4,901), and 1 during the 2002-2003 school year in Matamoros, Tamaulipas, Mexico (n = 669). We assessed disturbed sleep during the 4 weeks before the survey.

**Results:**

The prevalence of disturbed sleep in Matamoros was 36% and in the Lower Rio Grande Valley was 28%. Factors associated with disturbed sleep in both populations were smoking cigarettes, having ever used cocaine, having been forced to have sex, considering attempting suicide, feeling sad, and going without eating for 24 hours or more.

**Conclusion:**

This study revealed a high prevalence of disturbed sleep in high school students living on the Texas-Mexico border. This public health issue should be further investigated in both communities.

## Introduction

Disturbed sleep affects mental health, quality of life, and activity levels in adults and children (1,2). It affects students' learning ability, cognitive function, and behavior (3,4) and results in mood disturbance (2) and increased risk for accidents and injuries (4). Sleep problems are associated with personal and family psychosocial problems (5). Few studies, however, describe the prevalence of sleep problems among adolescents (4-9), and even fewer studies explore these problems among Hispanic adolescents (10).

People who live near the US-Mexico border are unique in their cross-acculturation. Studies have found that risk behaviors among border populations differ from those among nonborder populations (11-13). Since differences in governance, language, access to goods, and health care exist, we hypothesized that differences in the prevalence of disturbed sleep among adolescents on the Texas-Mexico border may also exist.

We estimated the prevalence of disturbed sleep among adolescents living on both sides of the Texas-Mexico border. We explored the relationship between disturbed sleep and demographic characteristics, indicators of mental health functioning, violence, obesity, and unhealthful eating. We also estimated the prevalence of sleep patterns and problems related to sleep among adolescents in the city of Matamoros, Mexico, by sex because sex differences have been observed among adolescents for lifestyle and behavioral risk factors (14,15).

## Methods

### Setting, design, and participants

The Lower Rio Grande Valley (LRGV) comprises 4 counties in Texas on the Mexico border: Cameron, Hidalgo, Starr, and Willacy. LRGV had 978,369 residents in 2000; residents are predominantly Hispanic (88%), of Mexican American origin (86% of Hispanics), and low income (35% below the poverty threshold in 1999) (16). Brownsville is the county seat of Cameron County; its sister city in Mexico is Matamoros in the state of Tamaulipas. Matamoros is less than 3 miles south of Brownsville and in 2000 had a population of 412,544 (17).

Data for this study came from 2 school-based surveys. The first survey (18) was conducted in English during the 2000-2001 school year in LRGV. This survey was then translated into Spanish, back-translated into English, and cross-culturally adapted for Mexican nationals. This second survey was conducted during the 2002-2003 school year in Matamoros (12). A full description of both surveys, study designs, participants, and samples has been reported previously (12,18). Briefly, ninth-grade students from both sides of the Texas-Mexico border were surveyed to determine risk behaviors that contribute to illness and death among adolescents. Both surveys were self-administered during regularly scheduled classes. Passive parental consent was obtained in LRGV and active parental consent was obtained in Matamoros; the children provided consent in both surveys. Survey protocols were reviewed and approved by the University of Texas Health Science Center at Houston's Committee for the Protection of Human Subjects and by the Secretariat of Education of the state of Tamaulipas.

All ninth-grade students from randomly selected schools in LRGV were invited to participate. The LRGV sample included 4,901 students, all of Hispanic origin, from 13 of 18 high schools; this sample represented 23% of ninth-grade students in LRGV during the 2000-2001 school year. In Matamoros, we randomly selected 8 of 28 high schools. All ninth-grade students from selected schools were invited to participate in the survey. The Matamoros sample included 669 students and represented 12% of ninth-grade students in Matamoros during the 2002-2003 school year. The response rates for the surveys were 67% from the LRGV and 58% from Matamoros. We did not collect data on students who did not participate.

### Surveys

The surveys asked 7 questions about sleep patterns and problems related to sleep. These questions were used previously to operationalize *Diagnostic and Statistical Manual of Mental Disorders, Fourth Edition* (19), symptom criteria for sleep disturbance (5,6). Two questions were related to amount of sleep reported in the past 4 weeks on weekday and weekend nights. Restorative sleep was assessed with the question, "During the past 4 weeks, how often have you felt really rested when waking up in the morning?" Overall quality of sleep was assessed with the question, "During the past 4 weeks, how would you rate the quality of your sleep overall?"

Three questions assessed problems related to sleep disturbance: “During the past 4 weeks, how often would you say you have had any of these problems related to your sleep: 1) trouble falling asleep (difficulty initiating sleep), 2) waking up in the middle of the night and finding it hard to get back to sleep (difficulty maintaining sleep), and 3) waking up very early and not being able to get back to sleep (early morning awakening)?” An adolescent who experienced at least 1 of these problems often or almost every day in the past 4 weeks was defined as having disturbed sleep.

We analyzed demographic covariates as potential risk factors for disturbed sleep among adolescents; these covariates included sex, age, ethnic origin, employment status, and perceived family standard of living (used as a proxy for socioeconomic status). Ethnic origin was assessed in the LRGV cohort with the question, "If you are Hispanic or Latino in origin, are you Mexican American (born in United States), Mexican American (born in Mexico), Mexican national, or other Hispanic or Latino (Cuban, Central American, Puerto Rican, etc)?" Because of regulations, we could not ask about country of origin in Matamoros, and all students were considered to be Mexican nationals. Perceived family standard of living was collapsed into 3 categories: very well off, living comfortably or just getting along, and nearly poor or poor.

As an indicator of mental health, the survey also included questions on substance use. We asked about the frequency of smoking cigarettes, drinking alcohol, and using marijuana in the past 30 days. We also asked about lifetime use of cocaine and steroids. To measure mood disturbance, we asked, "During the past 12 months, did you ever feel so sad or hopeless almost every day for 2 weeks or more in a row that you stopped doing some usual activities?" Attempting suicide was measured with the question, "During the past 12 months, did you ever seriously consider attempting suicide?"

LRGV students self-reported their height and weight, and Matamoros students had their height and weight measured to the nearest 1 mm and 0.1 kg, respectively, while the students were wearing light clothing and no shoes. Body mass index (BMI) was calculated by dividing weight in kilograms by the square of the height in meters. We used BMI-for-age growth charts (http://www.cdc.gov/growthcharts/) to classify students as underweight or normal weight (<85th percentile), overweight (≥85th percentile to <95th percentile), or obese (≥95th percentile).

We assessed violence by asking whether students had been in a physical fight in the past 12 months. We also asked whether they had ever been forced to have sex. We measured 1 unhealthy dietary behavior by asking, "During the past 30 days, did you go without eating for 24 hours or more to lose weight or to keep from gaining weight?"

### Statistical analysis

Both surveys used a multistage stratified cluster design combined with probability proportional to school size sampling. Sampling weights were used in the analysis to account for the multistage stratified sampling design. In Matamoros, nonresponse adjustment and ratio adjustment for population-fixed totals were used from school records to ensure that the sex composition of the sample was the same as that of the total school enrollment. Adjustment was not done in the LRGV survey because of limitations in the sampling frame. Weighted percentages, means, standard errors, and test statistics were calculated with SUDAAN version 9.0 (RTI International, Research Triangle Park, North Carolina). Multiple logistic regression was used to estimate adjusted odds ratios with their corresponding 95% confidence intervals. Differences were considered significant at *P* < .05.

## Results

The median age of participants was 15 years in LRGV and 14 years in Matamoros. In LRGV, boys were more likely than girls to be above the 85th percentile of BMI for age. In Matamoros, the difference in BMI for age between boys and girls was not significant. Perceived family standard of living differed significantly between Matamoros and LRGV, and the difference was also significant between boys and girls in both areas.

In Matamoros boys reported sleeping fewer hours and having fewer sleep problems than did girls (Table 1). Students from Matamoros reported more hours of sleep on weeknights (weeknight median, 7.3; 95% confidence interval [CI], 6.9-7.7; weekend median, 7.7; 95% CI, 7.5-7.9) than did students from LRGV (weeknight median, 6.7; 95% CI, 6.6-6.8; weekend median, 7.0; 95% CI, 8.6-7.1) (*P* = .04).

The prevalence of disturbed sleep was 36% in Matamoros and 28% in LRGV (*P* = .01). The prevalence of disturbed sleep differed by sex in LRGV but not in Matamoros (Table 2). The prevalence of disturbed sleep did not differ by age in either area.

After adjusting for demographic characteristics, adolescents in LRGV who had a job were less likely to have disturbed sleep (Table 3). Except for BMI category, all the other factors were positively associated with disturbed sleep among adolescents in LRGV. Findings were similar in Matamoros, although the associations with having a job, drinking alcohol, using marijuana, using steroids, being overweight, and having been in a fight did not reach significance.

## Discussion

We found a lower prevalence of disturbed sleep in LRGV adolescents (28%) than in Matamoros adolescents (36%). These prevalences were higher than reported in adolescents in 4 European countries (26%) (7) but lower than in the north of France (40%) (8). Approximately 15% of the adolescents in Matamoros reported that they had difficulty falling asleep often or almost every day in the past month, which was similar to findings in LRGV adolescents (16%) (18) and New Zealand students (15%) (20). The prevalence of early awakening in Matamoros students was higher (25%) than in other studies (7,9,18).

Many of the factors we found to be associated with disturbed sleep have been identified in adult populations (21), but few data are available on adolescents (6,18). Adolescents in LRGV who had a job reported that they were less likely to have disturbed sleep, and to our knowledge no other studies have reported this association. Unfortunately, we do not have qualitative data to explain the reasons for this finding. Our finding of cocaine use as a risk factor for disturbed sleep among adolescents in both Matamoros and LRGV is consistent with other studies on insomnia (7).

The association we found between obesity and disturbed sleep in Matamoros is consistent with previous reports that inadequate sleep time may contribute to obesity in adolescents (22,23). An association between sleep disorders and eating disorders has been reported (24). We observed this association in both Matamoros and LRGV, where students who reported unhealthy weight loss were twice as likely to have disturbed sleep.

Although a previous study found an association between sleep problems and stress (9), to our knowledge, this is the first study to report an association between forced sexual intercourse and disturbed sleep in adolescents. Services and intervention programs are needed for victimized children. In addition to their other benefits, such intervention programs have the potential to lessen the negative health effects of disturbed sleep.

### Limitations

Our study has several limitations. First, our measure of disturbed sleep was not inclusive. We investigated hours of sleep, restorative sleep, and other symptoms of disturbed sleep, but we did not examine parasomnias or formal diagnostic criteria for insomnia (19). Second, we relied on self-reported data, although these are widely used in community surveys and correlate well with data obtained by objective measures of disturbed sleep (19,25). A third limitation was that our sleep items asked whether participants experienced symptoms of disturbed sleep almost every day for the past 4 weeks. Therefore, we could not differentiate between students with chronic versus acute sleep problems. A fourth limitation was conducting the survey in Matamoros 2 years after the survey was conducted in LRGV; however, results should be comparable because of the lack of interventions during those 2 years. A fifth limitation of our surveys is that we did not measure any chronic diseases, which may influence the sleep process (6).

### Conclusions

The challenge of overcoming the high prevalence of disturbed sleep among adolescents on the Texas-Mexico border region is immense. Teaching adolescents about healthy sleep habits may reduce this prevalence. Nevertheless, studies are needed that identify additional risk factors and potential strategies to reduce disturbed sleep among adolescents.

## Acknowledgments

This research was supported by a grant from the National Institutes of Health National Center on Minority Health and Health Disparities (NIH CMHD P20MD000170-019001). We thank the principals, coordinators, teachers, and other staff in the schools in Matamoros and LRGV for their support of this study. We also thank the students from the associate degree program in nursing at the University of Texas at Brownsville, the staff from Healthy Communities of Brownsville, Inc, the staff from the University of Texas Health Science Center at Houston School of Public Health Brownsville Regional Campus, and volunteers and staff members from the Secretaría de Educación de Tamaulipas. We also thank the reviewers for their helpful comments that improved an earlier version of this manuscript.

## Figures and Tables

**Table 1:** Disturbed Sleep Among 669 Adolescents in Matamoros, Tamaulipas, Mexico, 2003

Variable	Boys, %^a^	Girls, %^a^	Total, %^a^	Pearson χ^2^ b	*P* Value
**Hours slept weekday nights**					
≤6	22	15	18	5.11	.02
7	25	24	24		
8	27	28	28		
≥9	26	33	30		
**Hours slept weekend nights**					
≤6	25	19	22	0.29	.59
7	12	17	15		
8	18	20	19		
≥9	45	44	45		
**Frequency of restorative sleep**					
Rarely or never	4	6	5	0.83	.36
Sometimes	54	55	55		
Often	21	22	22		
Almost every day	20	17	18		
**Overall quality of sleep**					
Very good	15	12	14	2.64	.11
Fairly good	44	40	42		
Fairly bad	38	43	41		
Very bad	3	4	4		
**Difficulty initiating sleep**					
Rarely or never	50	43	46	0.14	.70
Sometimes	34	43	39		
Often	12	10	11		
Almost every day	4	3	4		
**Difficulty maintaining sleep**					
Rarely or never	56	39	46	11.43	<.001
Sometimes	36	50	44		
Often	7	7	7		
Almost every day	1	5	3		
**Early morning awakening **					
Rarely or never	46	37	41	4.65	.03
Sometimes	33	35	34		
Often	11	14	13		
Almost every day	9	14	12		

a Percentages may not total 100 because of rounding.

b All *df* = 1.

**Table 2:** Prevalence of Disturbed Sleep^a^ by Selected Risk Factors Among Adolescents in LRGV (n = 4,901) and Matamoros (n = 669)

Risk Factor	LRGV			Matamoros		

	% With Disturbed Sleep	% Without Disturbed Sleep	*P* Value^b^	% With Disturbed Sleep	% Without Disturbed Sleep	*P* Value^b^
**Total**	28	72	NA	36	64	NA
**Sex**						
Male	25	75	<.001	36	64	.71
Female	32	68		35	65	
**Age, y**						
≤14	28	72	.87	34	66	.67
15	28	72		37	63	
≥16	28	72		33	67	
**Perceived family standard of living**						
Very well off	21	79	<.001	37	63	.91
Living comfortably or just getting along	29	71		35	65	
Nearly poor or poor	51	49		36	64	
**Ethnic identity**						
Mexican national	24	76	<.001	36	64	NA
Mexican American (born in United States)	30	70		NA	NA	
Mexican American (born in Mexico)	21	79		NA	NA	
Other Hispanic or Latino	40	60		NA	NA	
**Have a job currently**						
Yes	27	73	.02	38	62	.55
No	32	68		34	66	
**Hours slept weekday nights**						
≤6	40	60	<.001	50	50	.006
7	24	76		38	62	
8	20	80		28	72	
≥9	23	77		33	67	
**Hours slept weekend nights**						
≤6	32	68	<.001	49	51	<.001
7	30	70		38	62	
8	25	75		29	71	
≥9	25	75		30	70	
**During the past 4 weeks, how often have you felt rested when waking up in the morning?**						
Rarely or never	38	62	<.001	63	37	<.001
Sometimes	22	78		40	60	
Often	25	75		35	65	
Almost every day	29	71		15	85	
**During the past 4 weeks, how would you rate the quality of your sleep overall?**						
Very good	12	88	<.001	14	86	<.001
Fairly good	20	80		30	70	
Fairly bad	47	53		45	55	
Very bad	66	34		71	29	
**Smoked cigarette in past 30 days**						
Yes	38	62	<.001	45	55	.05
No	24	76		33	67	
**At least 1 drink of alcohol in past 30 days**						
0 days	23	77	<.001	32	68	.07
1-5 days	30	70		42	58	
≥6 days	42	58		61	39	
**Marijuana use in past 30 days**						
0 times	25	75	<.001	36	64	.62
1-2 times	34	66		26	74	
≥3 times	42	58		55	45	
**Ever use cocaine**						
Yes	42	58	<.001	74	26	.01
No	25	75		35	65	
**Ever use steroids**						
Yes	43	57	<.001	52	48	<.001
No	27	73		35	65	
**Felt sad or hopeless every day for ≥2 weeks in a row**						
Yes	45	55	<.001	48	52	<.001
No	22	78		31	69	
**Considered attempting suicide**						
Yes	48	52	<.001	51	49	.05
No	24	76		33	67	
**Body mass index category^c^**						
Underweight/normal	28	72	.83	34	66	.001
Overweight	29	71		35	65	
Obese	28	72		41	58	
**In a physical fight in past 12 months**						
Yes	33	67	<.001	39	61	.21
No	26	74		33	67	
**Ever forced to have sex**						
Yes	45	55	<.001	70	30	.01
No	27	73		34	66	
**Gone ≥24 hours without eating in past 30 days**						
Yes	43	57	<.001	46	54	.04
No	25	75		34	66	

Abbreviations: LRGV, Lower Rio Grande Valley; NA, not applicable.

a Defined as difficulty initiating sleep, difficulty maintaining sleep, or early morning awakening often or almost every day in the past 4 weeks.

b Pearson χ^2^.

c Underweight or normal weight, <85th percentile; overweight, ≥85th percentile to <95th percentile; obese, ≥95th percentile.

**Table 3:** Odds of Disturbed Sleep by Selected Risk Factors Among Adolescents in LRGV (n = 4,901) and Matamoros (n = 669)

Risk factor	Odds Ratio (95% Confidence Interval)^a^		

	LRGV	Matamoros	Combined
**Have a job**			
No	1 [Reference]	1 [Reference]	1 [Reference]
Yes	0.79 (0.62-0.99)	1.18 (0.83-1.67)	0.88 (0.70-1.12)
**Smoked cigarette in past 30 days**			
No	1 [Reference]	1 [Reference]	1 [Reference]
Yes	1.93 (1.60-2.33)	1.64 (1.02-2.65)	1.91 (1.59-2.29)
**Drank alcohol in past 30 days**			
0 days	1 [Reference]	1 [Reference]	1 [Reference]
1-5 days	1.36 (1.15-1.61)	1.49 (0.57-3.94)	1.43 (1.08-1.89)
≥6 days	2.46 (1.73-3.50)	2.86 (0.63-12.90)	2.58 (1.86-3.57)
**Used marijuana in past 30 days**			
0 times	1 [Reference]	1 [Reference]	1 [Reference]
1-2 times	1.55 (1.24-1.93)	0.64 (0.21-1.96)	1.52 (1.20-1.93)
≥3 times	2.10 (1.43-3.07)	2.21 (0.44-11.10)	2.14 (1.49-3.06)
**Ever used cocaine**			
No	1 [Reference]	1 [Reference]	1 [Reference]
Yes	1.98 (1.42-2.78)	5.50 (2.00-15.13)	2.12 (1.56-2.89)
**Ever used steroids**			
No	1 [Reference]	1 [Reference]	1 [Reference]
Yes	1.96 (1.40-2.73)	2.24 (0.74-6.79)	1.99 (1.41-2.82)
**Felt sad or hopeless every day for ≥2 weeks in a row**			
No	1 [Reference]	1 [Reference]	1 [Reference]
Yes	2.76 (2.44-3.11)	2.16 (1.67-2.78)	2.63 (2.35-2.95)
**Considered attempting suicide in past 12 months**			
No	1 [Reference]	1 [Reference]	1 [Reference]
Yes	2.49 (2.08-2.98)	2.35 (1.24-4.45)	2.51 (2.00-3.16)
**Body mass index category^b^**			
Underweight/normal	1 [Reference]	1 [Reference]	1 [Reference]
Overweight	0.99 (0.84-1.16)	1.07 (0.77-1.49)	1.03 (0.89-1.21)
Obese	0.99 (0.85-1.17)	1.33 (1.04-1.69)	1.10 (0.97-1.25)
**In a physical fight in past 12 months**			
No	1 [Reference]	1 [Reference]	1 [Reference]
Yes	1.47 (1.20-1.80)	1.29 (0.91-1.84)	1.43 (1.18-1.73)
**Ever forced to have sex**			
No	1 [Reference]	1 [Reference]	1 [Reference]
Yes	2.07 (1.42-3.00)	4.44 (1.31-15.00)	2.25 (1.55-3.27)
**Gone ≥24 hours without eating in past 30 days**			
No	1 [Reference]	1 [Reference]	1 [Reference]
Yes	2.01 (1.42-2.85)	1.76 (1.23-2.51)	2.01 (1.52-2.67)

Abbreviation: LRGV, Lower Rio Grande Valley.

a All analyses are adjusted for sex, age, and perceived family standard of living. LRGV data are additionally adjusted for ethnic origin. Combined data are additionally adjusted for location (LRGV used as reference).

b Underweight or normal, <85th percentile; overweight, ≥85th percentile to <95th percentile; obese, ≥95th percentile.
